# The microRNA-455 null mouse shows dysregulated bone turnover

**DOI:** 10.1093/jbmrpl/ziaf007

**Published:** 2025-01-12

**Authors:** Lingzi Niu, Tracey E Swingler, Caterina Suelzu, Adel Ersek, Isabel R Orriss, Matthew J Barter, Dan J Hayman, David A Young, Nicole Horwood, Ian M Clark

**Affiliations:** Biomedical Research Centre, School of Biological Sciences, University of East Anglia, Norwich, NR4 7TJ, United Kingdom; Biomedical Research Centre, School of Biological Sciences, University of East Anglia, Norwich, NR4 7TJ, United Kingdom; Norwich Medical School, University of East Anglia, Norwich, NR4 7UQ, United Kingdom; Norwich Medical School, University of East Anglia, Norwich, NR4 7UQ, United Kingdom; Comparative Biomedical Sciences, Royal Veterinary College, London, NW1 0TU, United Kingdom; Institute of Genetic Medicine, Newcastle University, Newcastle-upon-Tyne, NE1 3BZ, United Kingdom; Institute of Genetic Medicine, Newcastle University, Newcastle-upon-Tyne, NE1 3BZ, United Kingdom; Institute of Genetic Medicine, Newcastle University, Newcastle-upon-Tyne, NE1 3BZ, United Kingdom; Norwich Medical School, University of East Anglia, Norwich, NR4 7UQ, United Kingdom; Biomedical Research Centre, School of Biological Sciences, University of East Anglia, Norwich, NR4 7TJ, United Kingdom

**Keywords:** microRNA, miR-455, null mouse, bone, FGF18

## Abstract

A wide range of specific microRNAs have been shown to have either positive or negative effects on osteoblast differentiation and function, with consequent changes in postnatal bone mass. A number of specific targets have been identified. We previously used CrispR-Cas9 to make a miR-455 null mouse, characterizing a behavioral phenotype with age. The current study identifies a bone phenotype, starting in younger animals. At 3 weeks of age, the miR-455 null mice (both male and female) display increased length of both long bones and vertebrae and, while this difference diminishes across 1 year, it remains significant. Increased bone formation in vivo is mirrored by an increase in osteogenesis from bone marrow–derived stem cells in vitro. This is accompanied by a decrease in osteoclastogenesis and osteoclast function. MicroCT analyses show increased trabecular bone and less porosity/decreased separation in the miR-455 null mouse, suggesting a more dense and stronger bone at 3 weeks of age; these differences normalize by 1 year. Gain-of-function and loss-of-function datasets show that *FGF18* expression is regulated by miR-455 and *FGF18* was validated as a direct target of miR-455. The regulation of *FGF18* by miR-455 is a likely mediator of its effect on bone.

## Introduction

The mutation or deletion of Dicer, an RNase, prevents the biogenesis of the majority of microRNAs, and studies on Dicer null mice demonstrated a strong role for microRNAs in skeletal development. Conditional knockout of Dicer in limb mesenchyme at the early stages of embryonic development leads to the formation of a smaller limb.^1^ Dicer null growth plates show a lack of chondrocyte proliferation but also enhanced hypertrophy.^1^ Conditional knockout of Dicer in chondrocytes results in skeletal growth defects and premature death.^2^ Conditional Dicer knockout in osteoprogenitor cells or in osteoblasts themselves can lead to either a postnatal decrease or increase in bone depending on which cells are targeted and the timing of the deletion.^3–5^ Deletion of Dicer in osteoclasts leads to an increase in postnatal bone mass.^6^^,^^7^ A wide range of specific miRNAs have been shown to have either positive or negative effects on bone cell differentiation and function, with consequent changes in postnatal bone mass, with a number of specific targets identified.^8^

MicroRNA-455 (miR-455) is genomically located in an intron of *COL27A1*, which encodes collagen type XXVII. We discovered that miR-455 is co-regulated with miR-140 in both ATDC5 and human mesenchymal stem cell (hMSC) models of chondrogenesis^9^^,^^10^ and is highly Sox9 inducible.^9^ miR-455-3p has been shown to act in early chondrogenic differentiation via direct targeting of *RUNX2*^11^ and potentially *HDAC2* and *HDAC8*.^12^ It may also impact on DNA methylation during chondrogenesis via *DNMT3A*.^13^

Two groups, as well as ours, have recently reported on an miR-455 null mouse.^14–16^ Ito et al^15^ found no differences in skeletal formation at P2 on skeletal prep with microCT analysis at 8 weeks old, showing no significant difference in bone volume, bone mineral density, and microarchitecture of the femur. Articular cartilage degeneration was not seen at 2 months of age but was apparent at 6 months with an increase in catabolic gene expression. HIF-2a was identified as a direct target of miR-455, which mediated the cartilage phenotype.^15^ Hu et al^14^ reported on an miR-455 null mouse but with little detail given; however, cartilage thickness was decreased in 6-month-old mice. In Mao et al,^16^ the same mouse model showed similar cartilage loss at 10 months of age.

We reported that our miR-455 null mouse showed a behavioral phenotype at 14 months of age, with significant recognition memory deficit and a slight increase in anxiety.^17^ A similar cognitive phenotype in an miR-455 null mouse was described by Kumar et al,^18^ with the converse shown in a transgenic mouse overexpressing miR-455-3p. MicroRNA-455 null mice also showed a decreased lifespan, again with the converse shown in the transgenic line.^18^ These phenotypes were associated with mitochondrial function.^18^^,^^19^

Here, we report a bone phenotype in our miR-455 null strain with a significant difference in both femur and vertebral length at 3 weeks of age. Bone formation in the null mice increased in vivo, while osteogenesis from bone marrow–derived mesenchymal stem cells increased in vitro. MicroRNA-455 null cells also showed decreased osteoclastogenesis and osteoclast function.

## Materials and methods

### MicroRNA-455 null mouse

MicroRNA-455 null mice were made using CrispR-Cas9 by the Transgenic Unit, University of Manchester (https://sites.manchester.ac.uk/genome-editing-unit), and bred to the C57/BL6 background as described.^17^ A 35-base deletion (confirmed by sequencing) removes part of both miR-455-3p and miR-455-5p along with the intervening hairpin. MicroRNA-455 was not expressed in the null mice in any tissue tested, while expression of *Col27a1* was unaffected. All mouse experiments were performed in compliance with the ARRIVE (Animal Research: Reporting of In Vivo Experiments) guidelines (https://www.nc3rs.org.uk/arrive-guidelines) under license PF50C7689 granted from the Home Office (United Kingdom) in accordance with the guidelines and regulations for the care and use of laboratory animals outlined by the Animals (Scientific Procedures) Act 1986 according to Directive 2010/63/EU of the European Parliament. Protocols were approved by the Animal Ethics Committee of the University of East Anglia and the Home Office, United Kingdom.

### Measurement of bone length

Mouse tails and hind limbs were harvested at 3 weeks, 6 months, and 1 year, followed by fixation in 10% (wt/vol) NBF (Sigma-Aldrich, Merck) for 24 hours at room temperature and stored in 70% (vol/vol) ethanol at 4°C until scanned. Plain X-ray imaging was conducted using the Bruker In-Vivo Xtreme system (Bruker). Maximal axial length of caudal vertebrae and limb bones was measured using Fiji Image J. The femur length was defined by the distance between the most proximal/superior point of the femur head and the most distal point of the femoral condyle along the long axis. The tibia length was defined by the maximal distance between the medial tibial plateau and the tibial plafond along the long axis.

### MicroCT

Mouse hind limbs were dissected, fixed, and dehydrated, as mentioned above. After rehydration in PBS for 30 minutes, tibiae were scanned using a Skyscan 1174 (Bruker) with 0.5-mm aluminum filter, and settings of 50 kV, 800 mA, 8.28-μm camera pixels, 0.7° rotation step, and 2 frames averaging. Initial scans of trabecular and cortical bones were reconstructed using NRecon (Bruker) and analyzed with CTAn (Bruker) according to the manufacturer. A volume of interest (VOI) encompassing the 1.2-mm region distal to the growth plate was selected for trabecular bone, while a VOI of a further 0.6 mm was applied for cortical bone. Three-dimensional (3D) visualization of the tibiae was generated using Ctvol (Bruker).

### Calcein incorporation

Calcein green (Sigma-Aldrich, Merck) was intraperitoneally injected into mice at 20 mg/kg at 1 and 3 days after weaning at P21. Tibiae were dissected 2 days after final injection (P26), fixed with 10% (wt/vol) NBF, dehydrated in gradient ethanol, and embedded in methyl methacrylate. Embedded tibiae were sectioned longitudinally and imaged using fluorescence microscopy (DMI3000B; Leica) equipped with a megapixel digital color camera (DFC310 FX; Leica). Mineral apposition rate was calculated based on the average distance of 2 calcein-labeled layers of the cortical bone per day.

### Osteogenesis

Murine bone marrow stromal cells (BMSCs) were isolated from the long limb bones of 3–4-week-old mice and subcultured in DMEM supplemented with 10% (vol/vol) FBS and 1% (vol/vol) penicillin/streptomycin (Pen/Strep) (all from Gibco, ThermoFisher Scientific), adapted from the method described.^20^ Murine BMSCs at passage 3 were seeded in a 48-well plate at a density of 6 × 10^5^ cells/well for Alizarin Red S staining, and in a 96-well plate at 1.5 × 10^5^ cells/well for quantitative RT-PCR (qRT-PCR) and alkaline phosphate (ALP) assay. Experiments were performed in quadruplicate for each mouse. Osteogenesis was induced by osteogenic medium consisting of 100 nM dexamethasone (Sigma-Aldrich, Merck), 10 mM β-glycerol phosphate (Sigma-Aldrich, Merck), 50 μg/mL ascorbic acid 2-phosphate (Sigma-Aldrich, Merck), 10% (vol/vol) FBS, and 1% (vol/vol) Pen/Strep in high-glucose DMEM (Gibco, ThermoFisher Scientific). Osteogenic medium was changed every 3 days until the desired time points.

### Alizarin Red S staining

Alizarin Red S staining was performed for analyzing calcium deposition of murine BMSCs undergoing osteogenesis for 28 days. Staining and quantification methods were adapted from de Sousa Brito et al.^21^ Briefly, cells were washed with PBS and fixed in 4% (wt/vol) formaldehyde (Sigma-Aldrich, Merck) for 1 hour at room temperature. Fixed cells were then immersed in 40 mM Alizarin Red S solution (Sigma-Aldrich, Merck) for 30 minutes, washed in double-distilled H_2_O, and air dried. Ten percent (wt/vol) cetylpyridinium chloride was used to dissolve Alizarin Red S. Eluents were transferred into a 96-well plate. Absorbance was measured at 595 nm using the EnVision 2103 plate reader (PerkinElmer).

### Alkaline phosphate assay

A colorimetric ALP assay was conducted to measure ALP activity of osteogenic murine BMSCs. Cells were washed with PBS, followed by adding 20 μL double-distilled H_2_O and 100 μL alkaline phosphatase yellow (pNPP) liquid substrate system (Sigma-Aldrich, Merck). Plates were then incubated at 37°C for 20 minutes. Eighty microliters of stop solution composed of 0.2 M NaOH were added and mixed thoroughly. Absorbance was detected at 405 nm. The concentration of ALP was calculated with regard to a standard curve generated from a series of ALP solutions of known concentrations.

### Osteoclastogenesis

Isolation and culture for murine osteoclasts on dentine were carried out using adapted published methods.^22^^,^^23^ Nonadhesive bone marrow cells were collected and seeded at 1 × 10^5^ cells/well on sterile dentine discs in 96-well plates. Experiments were performed in quadruplicate for each mouse. Seeded cells were cultured in αMEM (Minimal Essential Medium, Gibco, ThermoFisher Scientific) containing 10% (vol/vol) FBS (Gibco, ThermoFisher Scientific), 1% (vol/vol) Pen/Strep (Gibco, ThermoFisher Scientific), and 100 ng/mL recombinant mouse M-CSF (R&D Systems, Bio-Techne) for 2 days. Medium was subsequently changed to αMEM supplemented with 10% (vol/vol) FBS (Gibco, ThermoFisher Scientific), 1% (vol/vol) Pen/Strep (Gibco, ThermoFisher Scientific), 20 ng/mL recombinant mouse M-CSF (R&D Systems, Bio-Techne), and 50 ng/mL recombinant mouse RANK ligand (R&D Systems, Bio-Techne) for 4 days. αMEM was then acidified by adding concentrated HCl to obtain a final concentration of 0.01 N. Cells were further incubated in the acidified αMEM for 3 days to achieve a basal level of resorption.

### Tartrate-resistant acid phosphatase assay

Cells were fixed using 10% (wt/vol) NBF for 1 hour, and tartrate-resistant acid phosphatase (TRAP) activity was measured using TRAP solution containing 0.2 mg/mL naphthol AS-MX phosphate, 1% (vol/vol) N,N-dimethylformamide, and 0.4 mg/mL Fast Red Violet LB (benzanilide) salt for TRAP activity, as described^24^ (all from Sigma-Aldrich, Merck). Osteoclasts, defined as TRAP-positive cells with 3 or more nuclei, were examined using Fiji Image J.

For histologic assessment, mouse knee joints were fixed in 10% (wt/vol) NBF, decalcified in 14% (wt/vol) EDTA, and eventually embedded in paraffin (all from Sigma-Aldrich, Merck). Frontal sections with 5-μM thickness were serially cut using microtome (HM355S; Microm). Sections were stained with TRAP solution described above and counterstained with 0.06% (wt/vol) Fast Green (Sigma-Aldrich, Merck). A TrapHisto program^25^ was applied for semi-automated quantification of osteoclasts in the primary spongiosa of tibiae.

### Resorption pits quantification

Cells were removed from the dentine discs in 1 M ammonium hydroxide (Sigma-Aldrich, Merck). Dentine discs were then stained in 0.5% (wt/vol) toluidine solution (Sigma-Aldrich, Merck), as described by Danks et al.^26^ At least 3 independent views were imaged and examined for each disc. The surface area of pits was measured using Fiji Image J.

### Cell lines

Osteosarcoma cells 143B were a kind gift from Dr Darrell Green, University of East Anglia (UEA). DF1 is a spontaneously immortalized chicken dermal fibroblast cell line (a kind gift from Prof. Andrea Munsterberg, UEA). The chondrosarcoma cell line SW1353 was derived from human chondrosarcoma of humerus and purchased from the American Type Culture Collection (ATCC). Cells were cultured in DMEM supplemented with 10% (vol/vol) FBS and 1% (vol/vol) Pen/Strep (Gibco, ThermoFisher Scientific) in a 37°C incubator with 5% (vol/vol) CO_2_.

### Target identification

Three RNASeq datasets were used for target identification, as described by Paddy.^27^ These were from (1) knee cartilage from WT vs miR-455 null mice at age 3 months (GSE274395), (2) SW1353 cells transfected with mimics or inhibitors of miR-455-3p vs negative controls (as described above, GSE276347), and (3) chick limb bud microinjected with miR-455-3p mimic vs control (GSE276509). Differentially expressed genes were ranked based on fold-change and q-values.

### Transient transfection and target validation

The 143B human osteosarcoma cells were seeded in 96-well plates at a density of 7 × 10^3^ cells/well. The next day, cells were transfected with 150 nM miR-455-3p mimic (hsa-miR-455-3p miRCURY LNA miRNA Mimic; Qiagen) vs a non-targeting negative control (Negative Control miRCURY LNA miRNA Mimic; Qiagen) using Lipofectamine 2000 (ThermoFisher Scientific). For transfection of miR-455-3p inhibitor (hsa-miR-455-3p miRCURY LNA miRNA Inhibitor; Qiagen) or inhibitor control (Negative control A miRCURY LNA miRNA Inhibitor Control; Qiagen), a final concentration of 75 nM was used. All microRNA mimics, inhibitors, and controls were from Qiagen. Transfected cells were further incubated for 48 hours and harvested for qRT-PCR, as previously described.^28^ Cells were lysed and total RNAs were reverse-transcribed using a Cell-to-cDNA II kit (Ambion, ThermoFisher Scientific), followed by real-time PCR performed with a PCR 7500 system (Applied Biosystems). Primers (Sigma-Aldrich, Merck) and Universal Probe Library IDs (Roche) are listed in Table S1.

For luciferase assays, 3′UTR luciferase reporters were constructed using the In-Fusion HD Cloning kit (TaKaRa Bio), where the 3′UTR of human mRNAs predicted to be targets of miR-455 were inserted downstream of the firefly luciferase reporter of the pmirGLO Dual-Luciferase miRNA target expression vector (Promega). Nonfunctional mutations were introduced to the miR-455 seed sites using the QuikChange Lightning Multi Site-directed Mutagenesis kit (Agilent). DF1 cells were transfected with 50 nM miR-455-3p mimic or non-targeting control (as described above) using Lipofectamine 2000 (ThermoFisher Scientific); 24 hours after miRNA transfection, cells were transfected with 100 ng 3′UTR luciferase reporters, and incubated for a further 48 hours. Firefly and Renilla luminescence were quantified using the Dual-Glo luciferase assay system (Promega) and a multilabel plate reader (EnVision 2103; PerkinElmer). Primers (Sigma-Aldrich, Merck) for subcloning and mutagenesis are listed in Table S2.

### Statistical analysis

Data were tested for normal distribution comparison between 2 means analyzed using Student’s *t* test. One-way ANOVA with post hoc Tukey’s test was used to compare between multiple samples using GraphPad Prism version 9.

## Results

### MicroRNA-455 null mice have increased bone length

**Figure 1:**
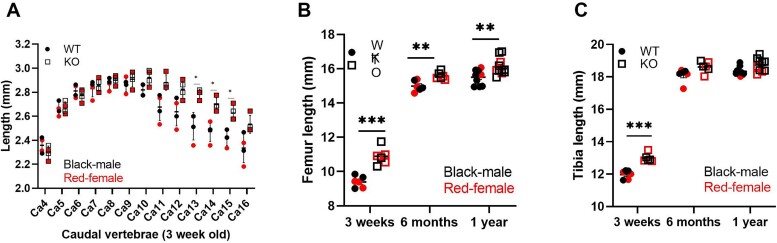
Measurement of bone length across age. A: Caudal (Ca) vertebrae were measured on X-ray and numbered from Ca4 (after lumbar vertebrae) (*n* = 4; *t* test with Benjamini-Hochberg correction). ^*^q < .05. Values are mean ± SEM. B and C: Lengths of tibia and femur were measured on X-ray (*n* = 12–20; *t* test with Benjamini-Hochberg correction). ^**^q < .01; ^***^q < .001.

We have previously shown that miR-455 null mice were significantly heavier than WT mice from around 6 months of age.^17^ The lengths of the caudal vertebrae (from Ca4 to Ca16) were measured by X-ray at 3 weeks of age (Figure 1A), with those from miR-455 null mice showing greater length (of Ca12 to Ca16) than WT mice. More distal caudal vertebrae showed a greater increase in length compared with the proximal vertebrae, with a similar pattern displayed at 6 months of age (data not shown). Femur and tibia lengths were measured using X-ray at 3 weeks, 6 months, and 12 months of age. Femur length was significantly greater in miR-455 null mice at all ages, although for tibia, it only remained significant at 3 weeks of age (Figure 1B, C). Although Figure 1 analyzes both sexes of mice, these are plotted separately in Figure S1, showing identical patterns. Measurement of the growth plate at 3 weeks of age showed a small statistically significant increase in overall size in miR-455 null vs WT mice, coming from an increase in the size of the resting zone and a decrease in the hypertrophic zone in the miR-455 null mice (both *p* < .05; Figure S2).

### MicroRNA-455 null mice exhibit greater bone deposition and osteogenesis

**Figure 2:**
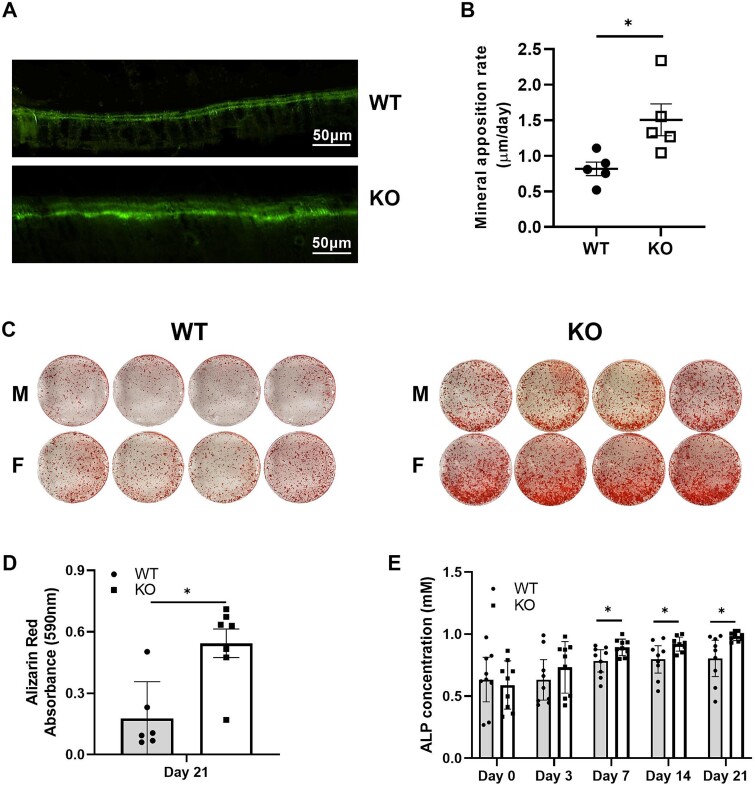
Osteoblast function and osteogenesis. A, B: Sequential intraperitoneal calcein injections were undertaken were undertaken 2 days apart with sacrifice 48 hours later. Bone was embedded in methyl methacrylate, sections visualized, and the amount of bone formed between the 2 injections quantified using image analysis (*n* = 5). Values are mean ± SEM. Student’s *t* test, ^*^p < .05. C: Bone marrow–derived stem cells were differentiated through osteogenesis across 28 days and calcium phosphate stained using Alizarin Red, extracted and quantified in panel D (*n* = 6; 3 males [M] and 3 females [F]. Values are mean ± 95% CI. Student’s *t* test, ^*^p < .05. E: Alkaline phosphatase (ALP) in the conditioned medium was quantified (*n* = 8; 4 males and 4 females). Values are mean ± 95% CI. Two-way ANOVA with Fisher’s least significant difference test, ^*^p < .05.

Bone formation in the mouse femur was assessed by double calcein labeling. Quantification of the matrix apposition rate showed significantly increased bone formation in the miR-455 null mice compared with WT mice (Figure 2A, B), demonstrating increased osteoblast function. Bone marrow–derived stem cells were extracted from the long bones of mice at P21 and differentiated into mature osteoblasts over 28 days. Alizarin Red staining demonstrated increased bone formation, with ALP activity in the medium increasing from day 7 (Figure 2C, D). Alkaline phosphatase staining increased in the miR-455 null mice compared with WT mice from day 7 onwards (Figure 2E). Bone marrow stromal cells were also differentiated through adipogenesis across 14 days and, although Oil Red O staining was greater in WT compared with miR-455 null mice, there was substantial cell death and the number of remaining adipocytes was low (data not shown). In order to support this, adipocyte ghosts were stained in the bone marrow of 3-week-old mice, showing that the number of adipocyte ghosts was decreased in the miR-455 null mice (Figure S3).

### MicroRNA-455 null mice exhibit decreased osteoclastogenesis

**Figure 3:**
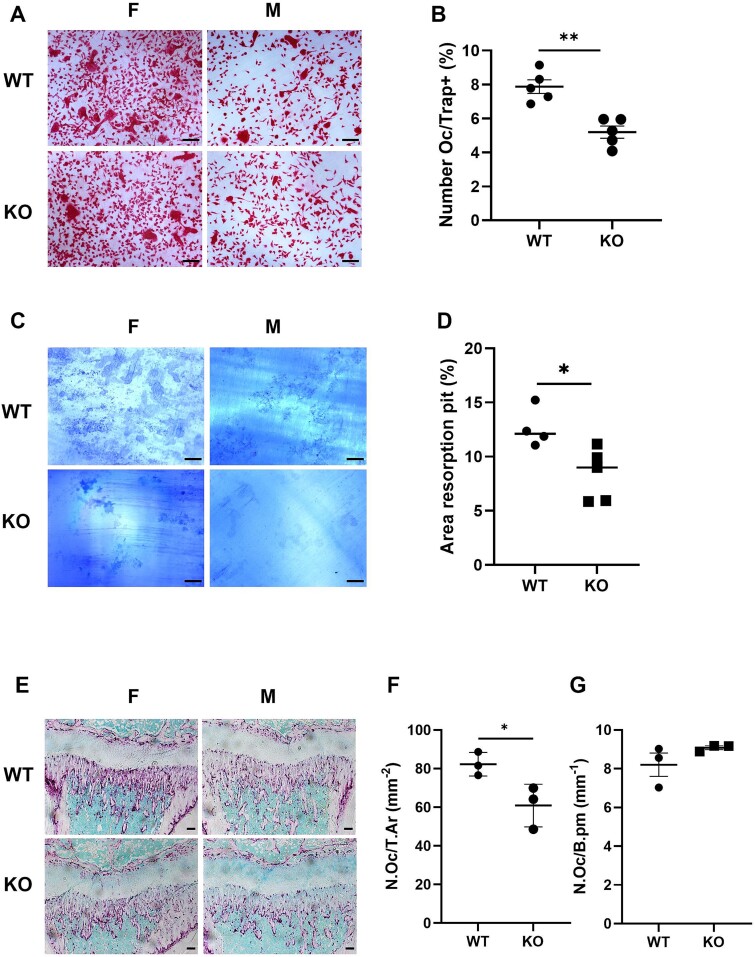
Osteoclastogenesis, osteoclast (Oc) number, and function. A, B: M-CSF–dependent bone marrow macrophages were cultured from the bone marrow of 3–5-week-old miR-455 null (KO) or WT mice and subjected to an osteoclastogenesis assay with cells cultured on dentine slices. TRAP staining identified and image analysis measured multinucleated osteoclasts (A, B), while Toluidine Blue (C, D) staining of dentine showed resorption pits. Values are mean ± SEM; *n* = 5. Student’s *t* test, ^*^p < .05, ^**^p < .01. TRAP staining in the distal tibia of 3-week-old mice showed osteoclasts in the primary spongiosa (E), with quantification expressed per unit area (F; N.Oc/T.Ar, number of osteoclasts per unit tissue area) or bone perimeter (G; N.Oc/B.pm, number of osteoclasts per unit bone perimeter). Scale bar = 100 μm.

The M-CSF–dependent bone marrow macrophages were cultured from the bone marrow of 3-week-old miR-455 null or WT mice and subjected to an osteoclastogenesis assay with cells cultured on dentine slices. TRAP staining identified a significant increase in multinucleated osteoclasts in WT cells compared with miR-455 null cells (Figure 3A, B), while Toluidine Blue staining also showed a significant increase in bone resorption (Figure 3C, D). This indicates that miR-455 knockout leads to decreased differentiation to osteoclasts, along with a potential decrease in function.

In vivo, TRAP staining in 3-week-old mice showed a significant decrease in TRAP-positive osteoclasts per unit area in the miR-455 null mice compared with WT mice. However, this difference was not apparent after normalization to bone perimeter (Figure 3E, F, G).

### MicroRNA-455 null mice show changes in both trabecular and cortical bone

**Figure 4:**
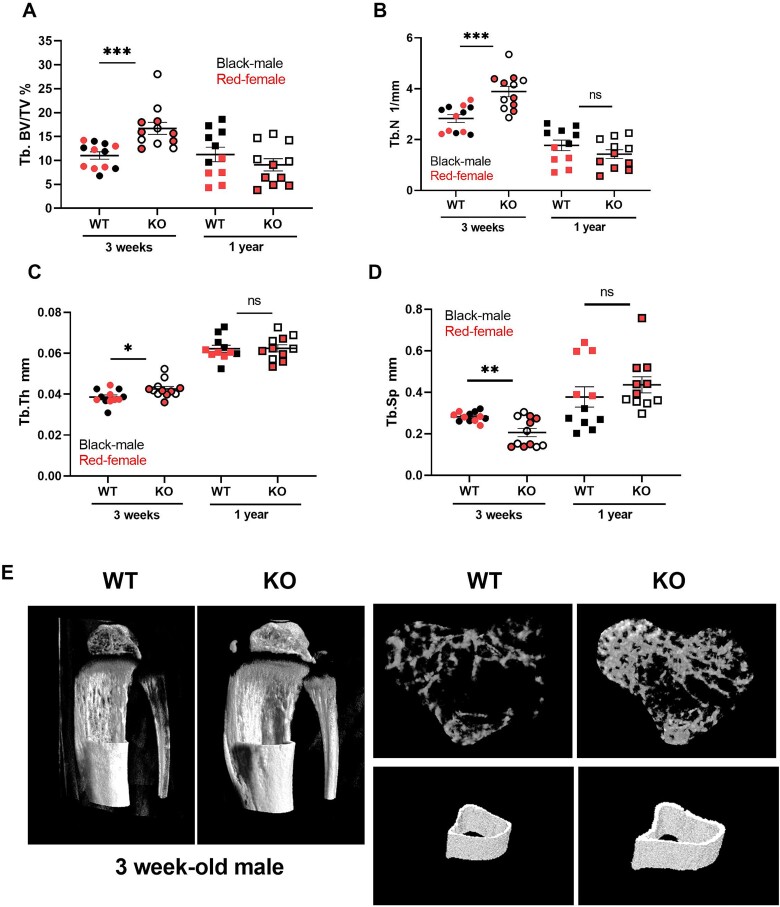
A–D: MicroCT analysis of miR-455 null mouse vs WT bone. Mouse hind limbs were dissected, fixed and dehydrated, and scanned using a Skyscan 1174 (Bruker) (see Materials and methods). Values are mean ± SEM; n = 11. Student’s t test, ^*^p < .05, ^*^^*^p < .01, ^*^^*^^*^p<.001. E: Three-dimensional visualization of the tibiae was generated using Ctvol (Bruker). Abbreviations: Tb.BV/TV%, percent trabecular bone volume; Tb.N, trabecular number; Tb.Sp, trabecular separation; Tb.Th, trabecular thickness.

MicroCT analysis showed that trabecular volume, as a percentage of total volume, increases in the miR-455 null mouse at 3 weeks old in both males and females (Figure 4A). Both trabecular number and thickness also increased in the miR-455 null mice at this age (Figure 4B, C). There was also a decrease in trabecular separation and therefore a decrease in porosity (Figure 4D) in the miR-455 null mice. All of the changes lost significance at 1 year of age and there was no difference between males and females of different genotypes (plotted together). These changes are mirrored for cortical bone (Figure S4). A reconstruction of bone features is shown in Figure 4E.

### *FGF18* is a direct target for microRNA-455

**Figure 5:**
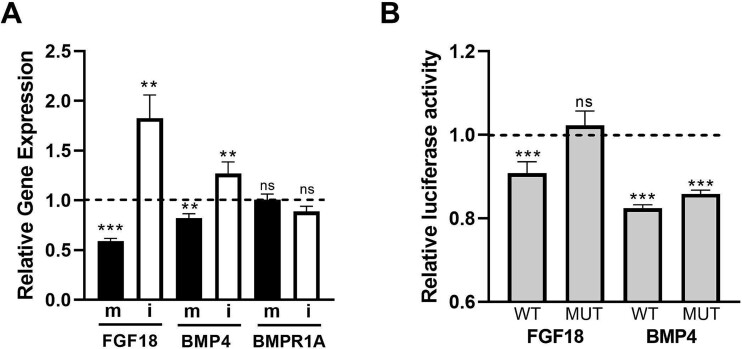
Identification and validation of relevant miR-455 targets. A: Human 143B osteosarcoma cells were transfected with 150 nM miR-455-3p mimic vs control or 75 nM miR-455-3p inhibitor vs control for 48 hours prior to measurement of gene expression by quantitative RT-PCR (qRT-PCR). Results are expressed as fold expression compared with control (dotted line). B: Chick DF1 fibroblasts were transfected with 100 ng 3′UTR of FGF18 or BMP4 in pmiRGLO, either WT sequence or with miR-455-3p targets mutated (mutant) in the presence 50 nM miR-455-3p mimic or non-targeting control for 48 hours prior to harvest and luciferase assay. Results are plotted relative to non-targeting control (dotted line). Values are mean ± SEM; *n* = 4. Student’s *t* test, ^**^p < .01, ^***^p < .001.

We had available a number of RNASeq datasets in which we explored the expression of genes relevant to the skeleton (see “Materials and methods”, although these were not datasets from bone or bone cells). Table 1 shows 12 genes that were increased in expression by more than 2-fold (*p* < .05) within articular cartilage taken from 3-month-old miR-455 null mice compared with WT mice with the addition of *Bmpr1a*, which has 9 predicted seed sites for miR-455-3p in its 3′UTR. We compared this with the expression in SW1353 cells overexpressing either miR-455-3p mimic or inhibitor and also chick limb bud microinjected with miR-455-3p mimic. The only gene, following the expected expression pattern (increased in miR-455 null, decreased by miR-455-3p mimic, and increased by miR-455 inhibitor), was *FGF18*, which is also predicted to be a direct target for miR-455-3p. *BMP4* and *BMPR1A* were also pursued, although *BMP4* has no seed sites for miR-455-3p in its 3′UTR. In 143B human osteosarcoma cells, *FGF18* and *BMP4* expression was decreased by overexpression of miR-455-3p mimic and increased by overexpression of miR-455-3p inhibitor (Figure 5A–C). No effect was seen on the expression of *BMPR1A*. The 3′UTR of the *FGF18* and *BMP4* genes was subcloned into pmIR-GLO (WT) and the seed site(s) for miR-455-3p mutated (mutant). Constructs were transiently transfected into chicken DF1 fibroblasts in the presence of either a control miR mimic or miR-455-3p mimic. Luciferase assays (Figure 5D, E) show that the 3′UTR of both genes is repressed by miR-455-3p mimic, but only *FGF18* is rescued by mutation of the seed site, implying that *FGF18* is a direct target for miR-455-3p, but *BMP4* is not.

**Table 1:** Gene expression across RNASeq data.

**Gene**	**Fold miR-455 null vs WT (knee cartilage)**	**Fold miR-455-3p mimic vs control (SW1353)**	**Fold miR-455-3p inhibitor vs control (SW1353)**	**Fold miR-455-3p mimic vs control (chick limb bud)**	**Predicted target miR-455-3p**
***Vcan***	2.09	0.70	0.98	0.91	N
***Alox15***	2.80	—	—	—	Y
***Penk***	2.01	—	—	0.83	N
***Srd5a1***	2.05	0.88	0.94	0.67	Y
***Col2a1***	2.03	—	—	1.04	Y
***Bmp4***	2.76	0.94	1.32	1.15	Y
***Bmpr1a***	1.42	1.02	0.84	0.87	Y
***Epyc***	4.27	—	—	1.31	N
***Fgf18***	2.69	0.56	6.20	0.99	Y
***Hjv***	3.44	1.68	0.71	0.67	Y
***Col9a1***	3.09	—	—	0.83	N
***Ifitm1***	2.07	0.83	0.89	—	N
***Panx3***	2.34	0.65	0.88	0.86	N

Knee articular cartilage was taken from 3-month-old miR-455 null mice compared wth WT mice; SW1353 cells overexpressed either miR-455-3p mimic or inhibitor vs control (50 nM); developing chick limb bud was microinjected with miR-455-3p mimic vs control (4 μL of 1 mM) at HH20-21 (HH, Hamburger Hamilton) and incubated for 24 hours.

Abbreviations: N, no; Y, yes.

## Discussion

The most detailed previous analysis of an miR-455 null mouse was performed by Ito et al.^15^ The authors did not show any difference in bone morphometry, either postnatally or at 8 weeks of age. Although their microCT analyses used only groups of *n* = 3 male mice, our data show that there would likely be a measurable difference in bone, even at that group size. Differences in the resolution of the microCT system used may also have an impact on measurement here. Ito et al also showed a difference in spontaneous cartilage destruction with age, which was apparent at 6 months. Our analyses of cartilage do not show such a difference out to 1 year of age (data not shown). However, in a pilot (*n* = 6) experiment of the DMM (destabilisation of the medial meniscus) model, we did see a statistically significant (*p* < .05) increase in OARSI (Osteoarthritis Research Society International) score in the miR-455 null mice compared with WT mice when all joint compartments were summed (Figure S5). We have reported the same behavioral phenotype in our miR-455 null mice as Kumar et al.^18^

In agreement with the literature, there was no significant difference in bone length between males and females within either genotype.^29^ The increase in long bone length measured in the miR-455 null vs WT mice was most apparent at 3 weeks of age, diminishing with age. At this early time point, there was a significant difference in growth plate height, but it is unclear if this is sufficient to explain changes in downstream bone length. However, this growth plate difference came from an increase in resting zone height and a previous detailed morphometric analysis showed that this correlated with bone length and bone growth rate in mice, whereas hypertrophic zone height did not.^30^

Both in vivo and in vitro, the miR-455 null mouse, or cells derived from it, showed effects on both bone formation (osteoblasts) and resorption (osteoclasts), with increased bone formation and decreased bone resorption in the miR-455 null mice. Data gained using cells from young (3-week-old) animals is consistent with the microCT data.

In microCT analyses, increased trabecular bone and less porosity/decreased separation would suggest a more dense and stronger bone, although this was not tested functionally. The difference between miR-455 null mice and WT mice in measures of microCT disappears by 1 year of age, despite the difference in long bone length remaining (although decreased). Interestingly, we have previously reported decreased expression of miR-455 in WT mice across age.^17^

In order to understand the molecular function of miR-455 in driving the bone phenotype, we made use of existing gene expression datasets in previous studies of cartilage, using tissue or cell models of genetic deletion of miR-455 or forced overexpression of inhibition. These were accessible datasets, rather than studies in the bone cells that would be more appropriate to the research question. Comparison of these datasets and analysis of potential miR-455 target sites in the 3′UTR of resultant genes led us to investigate *FGF18*, *BMP4*, and *BMP1RA* further. In a human osteosarcoma cell line, expression of *FGF18* and *BMP4* followed the same pattern as in the RNASeq data from SW1353 chondrosarcoma cells. Luciferase assays showed *FGF18* as a direct target of miR-455-3p; however, the repression of luciferase expression driven by the *BMP4* 3′UTR was not rescued by ablation of the miR-455 seed site. Either this gene is not a direct target for miR-455-3p or, further, cryptic miR-455 seed sites remain in the 3′UTR. Fgf18 has previously been shown to be required for osteogenesis in the mouse embryo,^31^ while the growth plate of the *Fgf18* null mouse is also elongated.^32^ Interestingly, overexpression or addition of *Fgf18* enhances osteogenesis from C3H10T1/2 cells.^33^ Osteoclasts do not express FGF18, although they may respond to it^34^; however, the mechanism of action of miR-455 in osteoclasts requires further work. While any microRNA likely has multiple gene targets that bring about functional effect, FGF18 is clearly a prime candidate to mediate the function of miR-455 on bone.

In summary, the miR-455 null mouse displays a bone phenotype underpinned by effects on both bone formation and resorption. FGF18 has been identified as a direct target for miR-455-3p, which may mediate this effect. Further research is needed to understand further the details of molecular pathways involved.

## Supplementary Material

Niu_et_al_miR455_and_bone_final_(supplemental_all)_ziaf007

## Data Availability

RNASeq data are available via the Gene Expression Omnibus (https://www.ncbi.nlm.nih.gov/geo/).
