# Supplementary material for: The microRNA-455 null mouse shows dysregulated bone turnover
Source: JBMR Plus. 2025 Jan 12;9(3):ziaf007. doi: 10.1093/jbmrpl/ziaf007 (PMC11831985; doi:10.1093/jbmrpl/ziaf007)
Supplement: Niu_et_al_miR455_and_bone_final_(supplemental_all)_ziaf007 [file niu_et_al_mir455_and_bone_final_(supplemental_all)_ziaf007.docx]

Supplementary Information

**Supplementary methods:**

Measurement of growth plate

Knee joints were harvested and fixed in 10% (v/v) neutral buffered formalin, followed by decalcification: 14% (w/v) EDTA for one week was used to decalcify 3 week old mice knees. 20% (v/v) formic acid for 24 hours was used to decalcify knees over 6 months old. After decalcification, mice knees were dehydrated in gradient ethanol and embedded in paraffin. Paraffin-embedded tissues were serially sectioned at an interval of 5μm with a thickness of 6μm. Sections were stained with 0.1% (w/v) Safranin O and counterstained with 0.06% (w/v) Fast Green solution. Different zones of the growth plate were defined in terms of cell morphology and staining. The resting zone was defined as the area on top of the growth plate with scattered cells. Beneath the resting zone, the proliferative zone was the region where columns of chondrocytes reside. Hypertrophic zones presented reduced Safranin O staining and enlarged chondrocytes.

Staining of adipocyte ghosts

Sections from 3 week old mice were stained with 0.1% (w/v) Safranin O, 0.06% (w/v) Fast Green, following the same method described above. During processing for paraffin embedding, lipids were dissolved, leaving holes in tissues, known as ‘adipocyte ghosts’.

Destabilisation of the medial meniscus

Ten-week-old male mice were anesthetized by inhalation of isoflurane (3% for induction and 1.5–2% for maintenance) in oxygen (1.5–2 liters/minute). All mice received a subcutaneous injection of buprenorphine (Alstoe Animal Heath) postsurgery. The mice were fully mobile within 4–5 minutes after withdrawal of isoflurane. DMM was performed as previously described (Glasson et al 2010)), and sham surgery consisted of capsulotomy only OA was scored by 2 individuals in a blinded manner, using a validated histologic scoring system, as described previously (Glasson et al 2010), and results were expressed as the summed score for al four joint components.

Supplementary Figure 1: Measurement of bone length across age separated by sex A: Caudal (Ca) vertebrae were measured on X-ray and numbered from Ca4 (after lumbar vertebrae), n=2, mean +/- range B, C: Length of tibia and femur were measured on X-ray, n=3-7, t-test with Benjamini-Hochberg correction, *, q<0.05; **, q<0.01

Supplementary Figure 2: Tibial growth plate measurement. Coronal sections from 3-week old mice were stained with Safranin O and Fast Green and thickness (A, C) and width (B, D) of resting zone (RZ), proliferative zone (PZ) and hypertrophic zone (HZ) was measured along the growth plate at a similar location using Image J.

Supplementary Figure 3: Adipocyte ghosts within the bone marrow of the distal tibiae of 3-week old mice. Sections were stained with Safranin O and Fast Green. Scale bar = 100µm.

Supplementary Figure 4: MicroCT analysis of miR-455 null mouse vs. wild-type bone. Mouse hind limbs were dissected, fixed and dehydrated and scanned using a Skyscan 1174 (Bruker), see Methods. A-D: Ct.BS, cortical bone surface area; Ct.BV/TV, percent cortical bone volume; Ct.Po, percent cortical porosity; Ct.Th, cortical bone thickness.

Supplementary Figure 5: Destabilisation of the medial meniscus in 12-week old male mice, wild-type (WT) vs. miR-455 knockout (KO), harvested at 10 weeks post-surgery (n=6). Sham surgery knees (n=3) were operated by opening the capsule but without cutting the medial meniscotibial ligament. Osteoarthritis scores, using the OARSI semi-quantitative scoring system (Glasson et al 2010) were assessed by two blinded observers and the summed score for all four joint compartments is shown.

Glasson S, Chambers M, Van Den Berg W, Little C. The OARSI histopathology initiative–recommendations for histological assessments of osteoarthritis in the mouse. Osteoarthritis and cartilage. 2010;18:S17-S23

Supplementary Table 1 Primers and UPL (Roche Universal Probe Library) probes for qRT-PCR

| **Gene** | **Forward 5’-3’** | **Reverse 5’-3’** | **UPL#** |
| --- | --- | --- | --- |
| *BMP4* | GGAGGAGGAGGAAGAGCAGA | CACTGGTCCCTGGGATGTTC | 31 |
| *FGF18* | TACCTGTGCATGAACCGCAA | GGCCGTGTAGTTGTTCTCCA | 60 |
| *BMPR1A* | TCAAACGTTTGCGGCCAATT | AATGCTGTGAGTCTGGAGGC | 107 |
| *VCAN* | TGAGGTTGCAACACCACCAT | TTTTGCAGCGATCAGGTCCT | 97 |
| *ALOX15* | TCAGGTTCCCTTGTTACCGC | CAGCTCTTCTTCCCGGTGTT | 124 |
| *SRD5A1* | GAGGCAAAGGCATCTGGACT | CACAGGCAGAGCAGCTTACT | 23 |
| *SFRP2* | ACCATTTCTGCTCCGGGATC | AGGAAATGCTGGGGATGCAA | 64 |

Supplementary Table 2 Primers sequences for In-Fusion cloning and site-directed mutagenesis.

| **3’UTR** | **Forward-SacI 5’-3’** | **Reverse-XhoI 5’-3’** | **Site mutation 5’-3’** |
| --- | --- | --- | --- |
| *BMP4* | TTGTTTAAACGAGCTCGATCAGGCAGTCCTTGA | CGACTCTAGACTCGAGACCTTTAAAATAATGACTCATTTTAT | CTCACCCACACACTACACAGACTGCTTCCTTATAGCT**CTCGAG**TTATTTAAAAAAAAAAAAAAAAAAGGAAAAAATCCCTA |
| *FGF18* | TTGTTTAAACGAGCTCCCACACTCACACTCCCAGAAA | CGACTCTAGACTCGAGTGTTGAGGCAGTAATACTTGGTT | GATGACAAAAGACTCACGCAAAG**CTCGA**GTAGTCAACCCACAGGTGCTTGTC |
| *BMPR1A* | TTGTTTAAACGAGCTCATATTGGTGGCCGGTGGTTT | CGACTCTAGACTCGAGTGCTTCCACACACCATTTGA | Site 1: GGCCAAAAGAAGTTTAAAGCATCTGTAAATTT**CTCGA**GTTTTCCTTCAACCACCATTTTTTTTGTGG  Site 2 and 3: CGCTCTGTCGCCCAGGCT**CTCGA**GCG**AG**CT**C**CAGTGGCGCAATCTCGGC |
| *VCAN* | TTGTTTAAACGAGCTCAGCCATAGGTGCAGTTTGCT | GACTCTAGACTCGAGTCTTTGGTGAAACAAAAGCAGAT | Site 1: TAATTTCAGTTTAGAGCAACTTGAAGAAGAGTAGACAAAAAATAAAA**CTCG**A**G**TAGAAAAAGAGAAAAAGGGCACAAAG  Site 2: TGATCTAGTAGGTTTCTATTTTTCCTTTCTCTTTACAA**CTCG**A**G**TAATACTTTCCTGTATTTATATCATAACGTGTATAGTG |
| *SRD5A1* | TTGTTTAAACGAGCTCTAGCCGGGAAAACTCCGTTC | CGACTCTAGACTCGAGAGCAAAAATTAAGTAGCATCTAA | GGAGTTGAAAACATGTCCACACAGAAAACCT**CTCG**A**G**GAATGTTTATAGCAGCTTTACTCAAAATTGC |
